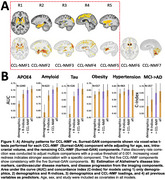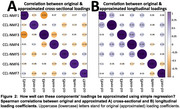# Dominant Trajectories of Aging‐related Brain Atrophy Identified in 48,949 Individuals via a coupled Cross‐sectional and Longitudinal Non‐negative Matrix Factorization

**DOI:** 10.1002/alz70856_101668

**Published:** 2025-12-24

**Authors:** Ioanna Skampardoni, Guray Erus, Ilya M. Nasrallah, Zhijian Yang, Kyunglok Baik, Haochang Shou, Konstantina Nikita, Christos Davatzikos

**Affiliations:** ^1^ Centre for Biomedical Image Computing and Analytics, University of Pennsylvania, Philadelphia, PA, USA; ^2^ School of Electrical and Computer Engineering, National Technical University of Athens, Athens, Attiki, Greece

## Abstract

**Background:**

Understanding brain aging heterogeneity is critical for early detection of neurodegeneration related to underlying neuropathologies and effective recruitment for clinical trials. Machine learning techniques have shown potential in this domain but often rely on cross‐sectional data, neglecting dynamic observations of pathological changes. This study applies Coupled Cross‐sectional and Longitudinal Non‐negative Matrix Factorization (CCL‐NMF), a novel framework integrating static and dynamic brain changes, to analyze aging‐related brain atrophy heterogeneity in a large, diverse dataset from 12 neuroimaging studies consolidated by the iSTAGING consortium (Skampardoni et al., 2024).

**Method:**

CCL‐NMF uses a mutually constrained NMF framework to identify brain aging components, combining population‐level aging effects from cross‐sectional data with individual‐specific dynamics from longitudinal data. Individual expression levels (loadings) for each component are estimated, optimizing the reconstruction of both data types to capture the interplay between static and dynamic aspects of brain alterations.

Structural MRI data from 48,949 individuals ≥50 years were analyzed. Comparative analyses were conducted against a weakly‐supervised generative adversarial network model, Surreal‐GAN (Yang et al., 2024), which relies solely on cross‐sectional data. Predictive performance for biomarkers, clinical variables, and disease progression was assessed using regression and Cox proportional hazards models with 5‐fold stratified cross‐validation.

To facilitate application to external datasets without retraining or harmonization, out‐of‐sample regression‐based estimation of CCL‐NMF loadings was implemented via NiChart (https://cbica.github.io/NiChart_Project/) with reliability validated through Pearson correlations with original loadings.

**Result:**

CCL‐NMF identified seven distinct brain atrophy components associated with Alzheimer's disease (AD), cognitive decline, and cardiovascular risk factors. Comparative analyses with the five Surreal‐GAN components revealed consistent representations between the two models, elucidating well‐reproducible brain atrophy components (Figure 1A). Importantly, CCL‐NMF provided a richer representation and demonstrated improved predictive performance across various outcomes, including AD, cardiovascular disease markers, and disease progression (Figure 1B). Furthermore, Spearman correlations between original and approximated CCL‐NMF loadings demonstrated high reliability, enabling seamless out‐of‐sample usage (Figure 2).

**Conclusion:**

CCL‐NMF offers a robust, interpretable framework for understanding brain aging and neurodegeneration by integrating cross‐sectional and longitudinal data. It outperforms cross‐sectional approaches, delivering richer representations with superior predictive accuracy, and supports easy application to external datasets through regression‐based loadings estimation under a web‐accessible server (neuroimagingchart.com).